# Manifestations of food protein induced gastrointestinal allergies presenting to a single tertiary paediatric gastroenterology unit

**DOI:** 10.1186/1939-4551-6-13

**Published:** 2013-08-06

**Authors:** Rosan Meyer, Catharine Fleming, Gloria Dominguez-Ortega, Keith Lindley, Louise Michaelis, Nikhil Thapar, Mamoun Elawad, Vijay Chakravarti, Adam T Fox, Neil Shah

**Affiliations:** 1 Department of Gastroenterology, Great Ormond Street Children’s Hospital, Great Ormond Street, London WC1N 3JH, UK; 2 Centre for Children’s Cancer and Blood Disorders, Sydney Children’s Hospital, Sydney, Australia; 3 Faculty of Medicine and Health and Life Science, University of Southampton, Southampton SO171BJ, UK; 4 Princess Alexandra Hospital NHS Trust, Harlow, Essex CM20 1QX, UK; 5 MRC & Asthma UK Centre in Allergic Mechanisms of Asthma, Division of Asthma, Allergy and Lung Biology, Guy’s and St Thomas’ NHS Foundation Trust, King’s College London, London SE1 7EH UK

## Abstract

**Background:**

Food protein induced gastrointestinal allergies are difficult to characterise due to the delayed nature of this allergy and absence of simple diagnostic tests. Diagnosis is based on an allergy focused history which can be challenging and often yields ambiguous results. We therefore set out to describe a group of children with this delayed type allergy, to provide an overview on typical profile, symptoms and management strategies.

**Methods:**

This retrospective analysis was performed at Great Ormond Street Children’s Hospital. Medical notes were included from 2002 – 2009 where a documented medical diagnosis of food protein induced gastrointestinal allergies was confirmed by an elimination diet with resolution of symptoms, followed by reintroduction with reoccurrence of symptoms. Age of onset of symptoms, diagnosis, current elimination diets and food elimination at time of diagnosis and co-morbidities were collected and parents were phoned again at the time of data collection to ascertain current allergy status.

**Results:**

Data from 437 children were analysis. The majority (67.7%) of children had an atopic family history and 41.5% had atopic dermatitis at an early age. The most common diagnosis included, non-IgE mediated gastrointestinal food allergy (n = 189) and allergic enterocolitis (n = 154) with symptoms of: vomiting (57.8%), back-arching and screaming (50%), constipation (44.6%), diarrhoea (81%), abdominal pain (89.9%), abdominal bloating (73.9%) and rectal bleeding (38.5%). The majority of patients were initially managed with a milk, soy, egg and wheat free diet (41.7%). At a median age of 8 years, 24.7% of children still required to eliminate some of the food allergens.

**Conclusions:**

This large retrospective study on children with food induced gastrointestinal allergies highlights the variety of symptoms and treatment modalities used in these children. However, further prospective studies are required in this area of food allergy.

## Introduction

In 2004 the World Allergy Organisation (WAO) published its revised nomenclature for food hypersensitivity which was followed in 2010 by the National Institute of Allergy and Infectious Disease (NIAID) guidance on the classification of food hypersensitivity [1,2]. According to the more recent NIAID guidance, a food allergy can be immunoglobulin E (IgE)-mediated, non-IgE mediated or a mixture of both IgE and non-IgE mediated [1]. Unlike the acute manifestations of IgE-mediated food hypersensitivity, slower developing non-IgE mediated gastrointestinal food allergic reactions may be difficult to identify. This is a problem which is compounded by a lack of straightforward tests to confirm a suspected diagnosis and the difficulties in performing food challenges in children that may take days to develop symptoms [3]. In addition, diagnostic criteria for many gastrointestinal allergic conditions are constantly evolving, in particular the criteria for diagnosing eosinophilic oesophagitis has changed significantly over the last 10 years [4]. As a result, there is paucity in prevalence, diagnosis and management data for this type of food allergic condition. However, it is known that a significant number of food allergic children below 1 year of age do present with vomiting, diarrhoea, constipation and abdominal pain and a delay in diagnosis can lead to impaired growth and development, and significantly impact on the parental quality of life [5-7]. The current gold standard test for diagnosing gastrointestinal food allergies remains the elimination diet, followed by the reintroduction of food to confirm improvement of symptoms on exclusion and their return on repeat exposure of the specific allergen. We therefore set out to describe a group of children with food induced gastrointestinal allergies, diagnosed according to above ‘gold standard’ from our paediatric gastroenterology unit, to provide clinicians with more information on typical presentation and an overview on nutritional management strategies and prognosis.

## Methods

This retrospective analysis was performed at Great Ormond Street Children’s Hospital Gastroenterology Department - a tertiary referral centre that reviews on average 6000 children with gastrointestinal disorders per annum and has a specialist interest in paediatric food protein induced gastrointestinal allergies. Patients were referred via secondary or tertiary centres with the majority coming from the London and South-East England region, which include a wide ethnic population. Ethics approval was obtained from Great Ormond Street Hospital Ethics Committee. All patient records from 2002–2009 that included the following search terms: allergy, reflux, eosinophilic oesophagitis, enteropathy, colitis, proctocolitis, constipation (defined as < 3 stools per week, Bristol stool chart) [8,9], diarrhoea (defined according to the World Health Organization criteria) [10], abdominal discomfort together with cow’s milk, soy, egg, diary, wheat and other food exclusions/eliminations, were considered for inclusion. If medical records included these search terms, they were reviewed individually and only included in the study if the documented medical diagnosis of gastrointestinal food allergies was confirmed by an elimination diet and deterioration of symptoms on reintroduction. The reintroduction of foods occurred at home with dietetic support, due to the delayed nature of the allergy, which was the usual hospital policy. Patients were excluded from the analysis if information on the diagnosis, management or symptomatology was ambiguous (i.e. no clear diagnosis, dietary management not documented).

All case notes were reviewed by one researcher according to a standard data collection sheet to avoid bias (Table 1). Symptoms at time of diagnosis, age of onset, age of diagnosis, atopic family history, concomitant and co-morbid allergic disease, dietary management and discharge from the gastroenterology department were recorded. All patients in the cohort received the standard therapeutic approach, which was policy of the gastroenterology department at the treating centre. This included; a full medical review, anthropometrical measurements (weight, height and head circumference if applicable) and individualised dietary advice. The elimination diet was determined through an allergy focused history and depending on the severity of symptoms either all common non-IgE mediated allergens were removed or they were removed sequentially. Follow-up occurred within 3 months as per standard practice and depending on their clinical status referred back to their local hospital.

**Table 1:** List of data collected from the medical notes

**Specific data collected from the medical notes**	**Yes**	**No**
Child still under care of tertiary gastroenterology unit?		
Does the child have atopic eczema?		
When did eczema start?		
Was the eczema treated?		
Did eczema improve when on the elimination diet?		
Does the child have asthma?		
When did asthma start?		
Was the asthma treated?		
Does the child have allergic rhinitis?		
Is there a family history of one of the following?		
Parents and siblings have any allergic rhinitis/asthma/eczema/ food allergies/ intolerances		
What is the diagnosis in the medical records?		
What foods were recommended to be eliminated from your child’s diet as part of their treatment and what hypoallergenic formula?		
Milk		
Milk and soya		
Milk, soya, egg		
Milk, soya, egg and wheat		
Milk, soya, egg, wheat and others		
What symptoms did the child have?		
Diarrhoea		
Constipation		
Vomiting		
Abdominal pain		
Flatus/bloating/abdominal distension		
Screaming/ Back arching after feeding/ related to food		
Food aversive behaviour/feeding difficulties		
Faltering growth/significant weight loss/poor weight gain		
Frequent respiratory and viral infections requiring general practitioner /paediatrician’s attention (> 1 infection per month and lasting longer compared to siblings)		

We were interested in the cohort’s current status and contacted parents or carers of identified patients, to establish the patient’s current allergy status and any changes over time of symptoms experienced were discussed (Table 2). Information on specific IgE blood testing, where available were documented as > or < 0.35 kU/L, as they have very limited diagnostic value in our non-IgE mediated population. Any other medical investigation pertaining to the diagnosis of gastrointestinal allergies were also documented when available. Data was analysed using SPSS version 20 (IBM, Chicago) and SAS/STAT Software (USA) and presented in frequencies, median (range) and where applicable mixed model analysis was applied (Chi-square test, Mantel-Haenszel Chi Square Test).

**Table 2:** Parent interview questionnaire

**Does your child have one or more of the following?**	**Yes**	**No**
Does anyone in your immediate family (parents and siblings) have any allergic rhinitis/asthma/eczema/ food allergies/ intolerances?		
What foods did you have to eliminated from your child’s diet as part of their treatment?		
Milk		
Soya		
Egg		
Wheat		
Other		
What food(s) is your child still required to avoid?		
Milk		
Soya		
Egg		
Wheat		
Other		
Was your child ever on a hypoallergenic milk formula during the treatment of their allergic condition? If so, which one?		
Amino acid formula (Neocate LCP™, Nutramigen AA™)		
Extensively hydrolysed formula ( Nutramigen Lipil 1/2™, Pepti Junior™, Pepti 1 or 2™)		
Soy formula (Infasoy™, Wysoy™)		
Other		
Does your child currently experience any of these?		
Diarrhoea?		
*Loose watery stools (Bristol stool chart) > 3 per day or more than usual.*		
Constipation?		
*Excessive straining, low frequency, hard stools as per Bristol stool chart.*		
Vomiting?		
*Constant unexplained vomiting often associated with abdominal pain.*		
Abdominal pain?		
*Chronic abdominal pain that affected daily functioning such as school and sleep.*		
Faltering growth/significant weight loss/poor weight gain?		
*Weight loss and/or suboptimal height for age or faltering growth before/during the*		
*allergy treatment*		
Food aversive behaviour?		
*Child persistently pushing food away, gagging on food, holding food in mouth, spitting or throwing food, and crying before and during meals.*		
Flatus/bloating/abdominal distension?		
*Gassy bloating of the stomach which extends stomach and feels hard to press on. Also excessive belching and flatus.*		
Screaming/ Back arching after feeding/ related to food?		
*Continuous screaming as infant associated with back arching and kicked their legs out straight.*		
Frequent respiratory and viral infections requiring GP attention?		
*Frequent respiratory and viral infections requiring general practitioner /paediatrician’s attention (> 1 infection per month and lasting longer compared to siblings)*		
Have any of these symptoms improved or are not experienced by your child since the treatment for food allergies?		
Do you think that your child has outgrown their food allergy?		

## Results

We identified 4860 medical records between 2002–2009 from the Gastroenterology Department that were eligible for inclusion, however only 615 patient records (12.6%) fulfilled the criteria for inclusion set out in the methodology. Data from 437 children (203 female and 234 male) were included in the analysis, whilst 178 cases were excluded due to incorrect contact details or refusal to partake in the study. From the patients included in the study, 237 (54.2%) were already discharged from the gastroenterology department for on-going review by their general practitioners (GP) and local paediatrician as the severity of the food allergy was no longer deemed to warrant tertiary clinical care.

**Figure 1:**
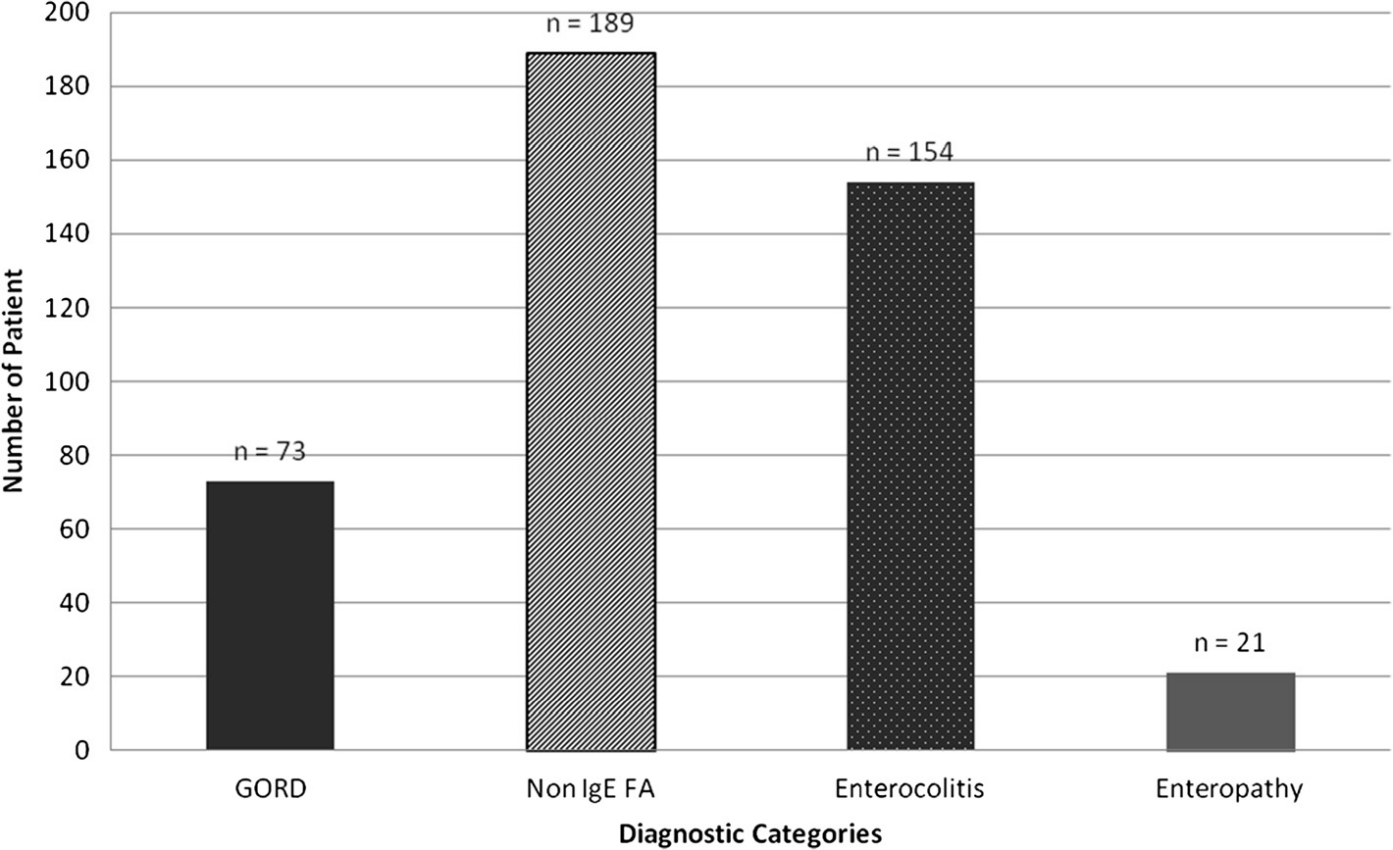
Diagnosis recorded in the medical notes (FA = Food Allergy, GORD = Gastro-oesophageal Reflux Disorder).

The median age of onset of symptoms of gastrointestinal allergy was 5 months (range 1–156 months). Patients were, on average only seen within our unit at 63 months (range 1–260 months) of age and at the time of performing this study, the median age of those children was 8 years. The most common diagnoses recorded in the medical notes was that of non-IgE mediated gastrointestinal food allergies (n = 189) and allergic enterocolitis (n = 154) (Figure 1). The majority (67.7%) of children had an atopic family history with parents or siblings with atopic disease: in 19.7% both were atopic (including eczema, asthma, hayfever or food allergy), 26.4% and 16.3% of mothers and fathers respectively and 5.3% of siblings. A significant association was found (p = 0.461) between having an atopic family history and the development of this type of gastrointestinal food allergies.

The most common symptoms recorded included abdominal pain and diarrhoea and are further summarised in Table 3. Although 54% had poor growth recorded as part of their medical history in the notes, the extend of growth failure (i.e. -1 or −2 z-scores) were not documented, as the majority were discharged to their local GP or paediatrician for follow-up and we did not have access to their medical records.

**Table 3:** Most common recorded symptoms

**Symptom**	**Frequency**
Abdominal Pain	89.9%
Diarrhoea	81%
Abdominal Distension/ Bloating	73.9%
Vomiting	57.8%
Weight loss	54%
Back-arching and screaming	50%
Constipation	44.6%
Rectal bleeding	38.5%

Two-hundred-and-fourteen (41.5%) patients had suffered from atopic dermatitis (AD) in our cohort. Of these, 79% developed features of AD prior to the age of 6 months and 21% after 6 months. The majority of the children who had asthma had developed symptoms before 4 years of age (56%), according to parental recall. This could not be verified by the gastroenterology medical records as many children were under the care of local general practitioners and paediatricians when diagnosed (Table 4). Many children (67.9%) had frequent respiratory infections recorded in their medical notes.

**Table 4:** Concomitant co-morbidities documented and experienced by parents (some had > 1 co-morbidities)

	**Number of patients**	**Percentage**
Atopic eczema	214	41.5
Asthma	140	32.1
Allergic rhinitis	197	45.1
Frequent respiratory infections	296	67.9

**Figure 2:**
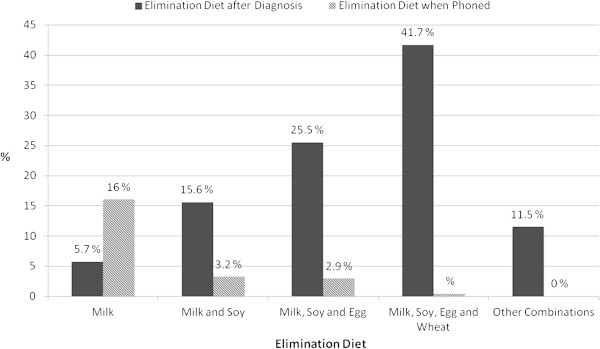
Elimination diets suggested by clinicians at diagnosis and foods still eliminated at follow-up phone call in 2009.

Patients were treated with elimination diets, as illustrated in Figure 2. The majority of patients were initially managed with a cow’s milk, soy, egg and wheat free diet (41.7%) and 11.5% eliminated these foods in addition to other foods to which they repeatedly reported symptoms. As many children were diagnosed at greater than 2 years of age, hypoallergenic formulae (i.e. extensively hydrolysed or amino acid formulae) were introduced in only 43.1% of children. Of those children that were prescribed a hypoallergenic formula, 53.4% had an amino acid formula, 23.3% an extensively hydrolysed formula and soya formula was used in 6.9%.

The telephone interview with the parents revealed that 24.7% of children (Figure 2), at the time of the interview (median age of 8 years) reportedly continued to react to some of the food allergens and therefore continued to avoid these. The combination of foods that continued to be eliminated varied, however the most common food/food combination from those still on elimination diets included: cow’s milk (16%), cow’s milk and soy (3.2%), cow’s milk, soy and egg (2.9%) and milk, soy, egg and wheat (0.4%). Some parents did however report that their children tolerated milk and/or egg in baked (extensively heated) foods (6.4%).

The median total IgE was 29.8 (Normal range for 3–6 years of age 0–71 KU/L) and IgA was 0.66 (Normal range for 3–6 years 0.4-2.0 g/L). Specific IgE results to food protein, are summarised in Table 5. In 295 (47.9%) of children, blood samples were sent for specific IgE, which included, milk, egg, soy and wheat. From these patients 88 (29.9%) had one or more IgE that was > 0.35 kU/l. No children in this analysis had skin prick testing or atopy patch testing to foods performed as it was unit policy only to use specific IgE.

**Table 5:** Specific IgE from the 88 patients that had 1 or more specific IgE > 0.35 kU/l

**Food allergen**	**Number of patients (%)**
Dairy	12 (14)
Dairy and egg	4 (5)
Dairy, soy and egg	22 (25)
Dairy, soy, egg and wheat	36 (40)
Above and other	14 (16)

## Discussion

Many studies have previously been published, describing specific gastrointestinal allergic conditions [11,12]. In particular, eosinophilic oesophagitis, allergic dysmotility, allergic constipation and food protein induced enteropathy have received a significant amount of attention [3,12-14]. To the best of our knowledge this is the largest review of this kind focusing on the symptoms, dietary management and prognosis of patients with food induced gastrointestinal food allergies.

This retrospective study focused on all types of non-IgE mediated gastrointestinal allergies, where food elimination resulted in resolution of symptoms. From all the patients seen in our tertiary gastroenterology department, 12.6% were identified as having the diagnosis of a food protein induced gastrointestinal allergy. Vieira et al. [15] found that 5.4% of children who attended various gastroenterology centres in Brazil had symptoms related to cow’s milk protein induced gastrointestinal allergies. The higher numbers of children with this type of allergy in the current study may be explained by the fact that children with all types of food protein induced gastrointestinal allergies were included, as opposed to children with only a cow’s milk allergy. In addition, the higher prevalence recorded in the current study may also be reflective of the fact that patients were being seen at a tertiary specialist referral centre. This is not representative of the population prevalence, which still remains elusive. Robust population prevalence studies for non-IgE mediated gastrointestinal food allergies are needed but at present numerous logistical challenges, related to the delayed nature of symptoms, which makes a double blind placebo controlled food challenge, the gold standard for diagnosis of food allergy, very difficult to perform.

Although onset of symptoms was reported on average at 5 months of age, our data indicated that patients were only seen within our gastroenterology department at a median age of 63 months. Our tertiary centre only accepts referrals from paediatricians, which implies that all children have previously had medical input, however they were referred onwards due to unsatisfactory symptom resolution with input or failure to recognise symptoms as being food related. A study by Sladkevicius et al. [16] in the UK found that it took on average 2.2 months for children with cow’s milk protein allergy, from initial consultation with the general practitioner to be commenced on a cow’s milk protein free diet. However this study was performed in primary care looking at both IgE-mediated and non-IgE mediated disease, the former of which is easier to recognise due to the quick onset of symptoms and therefore not comparable with our patient cohort. Although the delay in diagnosis is well recognised amongst specialists working with children with both IgE and non-IgE mediated food allergies, it has been better documented in the IgE-mediated population [16], whereas the data confirming a delay in the diagnosis of non-IgE mediated gastrointestinal allergies is not well studied. This is an important finding of this study, as there are numerous reports of nutritional deficiencies in children with IgE-mediated food allergies, where the diagnosis was delayed and/or parents were given inappropriate advice [17-19]. The psychological burden on families has also been well studied in IgE-mediated disease, which has been shown to be significant [20,21]. In addition, the prevalence of aversive feeding is high in this population of children and one could hypothesise that early recognition has the potential to reduce the incidence of nutrition deficiencies, the psychological burden and prevent some of the feeding difficulties they commonly experience. [22,23] The delay in diagnosis may also prompt parents to seek complementary and alternative therapy, which may be harmful for the child [24].

Our work also describes many symptoms that have in previous studies been linked to both mixed IgE and non-IgE gastrointestinal allergies [25]. These included vomiting, back-arching, constipation, diarrhoea, abdominal pain, abdominal distension/ bloating, rectal bleeding. What we have additionally highlighted, is the high prevalence of atopic dermatitis in these children (41.5%), which is similar (44%) to the findings of Latcham et al. [26] in a tertiary paediatric gastroenterology unit in the UK. A study by Caffarrelli et al. [27] focussing on children with atopic dermatitis, found that 42% of children with atopic dermatitis had gastrointestinal symptoms, ranging from vomiting, abdominal pain, diarrhoea to constipation. The same authors, in a separate study found that 28% of children with asthma had gastrointestinal symptoms [28]. Our study found that 32.1% of children diagnosed with gastrointestinal allergies also had asthma [28]. It is therefore important for dermatologists, allergists, gastroenterologists and pulmonologists to not forget the overlapping symptoms and ideally run a multidisciplinary clinic, where all these diagnoses are addressed in parallel [29].

Dietary management of non-IgE mediated gastrointestinal allergies is poorly described and studied in the literature. The choice of formula and elimination diets are often based on clinician preference or on the avoidance of the six versus four most common allergens, cow’s milk, soy, egg, wheat, (fish and nuts). Spergel et al. [30] found the most common foods involved in paediatric eosinophilic eosophagitis was milk, egg, wheat, soy, corn, beef, chicken, peanut, potato and rice, but the most common reported allergens involved were cows’ milk, egg and soy [30]. From our dietary elimination data, most children were managed on a four food elimination diet (cow’s milk, soy, egg and wheat) and in only very rare occasions (11.7%) other foods (strawberries, fish, citrus, yeast, sugars, preservatives) were excluded from the diet. As dietary elimination places a child at a significant risk of malnutrition, and cow’s milk, soy, egg and wheat contribute significantly towards nutritional intake, we always tried to challenge children with these foods, to avoid unnecessary avoidance. Other foods were only excluded based on consistent clinical history pointing towards a hypersensitivity reaction. Additionally, the elimination of foods depended on the severity of symptoms (including weight loss) and nutritional requirements of the child. As data has been collected in a tertiary gastroenterology department, the dietary restriction may reflect only those at the severe spectrum of food induced gastrointestinal allergies. What is known, is that the elimination of foods should be carefully considered as unnecessary avoidance of foods at an early age, may not only lead to nutritional deficiencies, but also an increase in feeding difficulties [31].

In this study the majority of children (53.4%) received an amino acid formula, which reflects the severity of patients seen within our tertiary centre. At the time of data collection, there were no UK- specific guidelines on formula choices for specific gastrointestinal conditions and the guidelines by the World Allergy Foundation had not been published [32].

A significant number of children according to parental reporting continued to require a dietary exclusion at 8 years of age. Although the natural progression of IgE-mediated allergy has been well described for most allergens [33,34], there is paucity of data for non-IgE mediated gastrointestinal allergies, with some data existing for cow’s milk protein allergy. Non-IgE mediated cow’s milk allergies has long been thought to have a better prognosis than IgE-mediated cow’s milk allergy [35,36]. However, many of those studies are outdated and may not reflect the same complexity of patients seen within our tertiary centre. In addition, the acquisition of tolerance was based on parental reporting and not food challenges, which may also explain why so many children continued to avoid some of the allergens. However more recent publications in IgE-mediated allergies have indicated a shift in acquiring later tolerance in cow’s milk protein allergy, which anecdotally is our experience as well in our population [33]. Further investigation of this is required in non-IgE mediated allergies, through a prospective longitudinal study methodology using the gold standard double blind food challenges as diagnostic confirmation.

Almost 68% of the allergic patient cohort had frequent upper respiratory tract infections, which was defined in this study as having more than 1 respiratory infection per month and lasting longer than siblings. Several studies have confirmed the numerous and prolonged severe respiratory infections in allergic children. Children with atopic dermatitis exhibit an increased susceptibility to bacterial, fungal and viral infections [37,38]. On our unit a previous study, had shown that 18% of the children had an associated detectable minor immunodeficiencies, which reflected in the recurrent upper respiratory infection including middle ear infections. Latcham et al. [26] also found in a retrospective study on children with food induced gastrointestinal allergies, that 45% had a serum IgA < 0.3 g/L. Many patients in this study had both gastrointestinal symptoms (i.e. GORD) as well as atopic dermatitis, which, in addition to the minor immunodeficiency seen in food induced gastrointestinal allergies, may explain our high incidence of respiratory infections.

This study has several limitations, including individual physician variation that may have influenced our results. We have made an attempt to account for this variation by using just one researcher to assess all medical records and to exclude any records where documentation may lead to ambiguity. Another limitation is that this study covered many years (2002–2009) of data. As the area of food allergy is generating a significant amount of research, it is plausible that the management of patients has significantly changed over the years. The results presented in this study represent a group with a variety of different gastrointestinal symptoms that have improved on the elimination of food from their diet and reproducibly worsened on their reintroduction. Although recorded, unlike other studies, we have not focused on specific gastrointestinal allergic disorders (i.e. food induced enteropathy, proctocolitis) as great inter-professional variation was seen in the diagnosis of these conditions, as shown by our results as well. This may be seen as a limitation of the study, however the spectrum of food allergies affecting the gastrointestinal tract leads to symptoms either due to the site of the inflammatory infiltrate such as in eosinophilic oesophagitis or transmural inflammation such as in eosinophilic ganglionitis, hence children often have overlapping symptoms that may imply the existence of more than one gastrointestinal allergic condition. This may have complicated the interpretation of data [39]. We therefore choose the success of food elimination and re-introduction as our entry criteria rather then specific diagnosis to reduce bias during data analysis. However the authors of this study acknowledge that the reintroduction of the offending allergens occurred at home and not in hospital. A double blind placebo controlled food challenge remains the gold standard, but for this population with delayed reaction of the severity seen in our tertiary centre, this was not feasible.

The fact that no child had allergy patch test may also be interpreted as a limitation. The role of patch testing in non IgE mediated and mixed reaction has been shown to be controversial, with different centres reporting contradictory findings [30,40]. As there was no consensus regarding the role of patch testing for all types of gastrointestinal food allergies (IgE and mixed) during 2002–2009 and the elimination diet followed by the reintroduction remained the most uncontroversial method. We do not think that the absence of this test is a significant limitation of this observational retrospective study. However, future prospective studies may benefit with the inclusion of this test.

## Conclusion

This large retrospective study on children with food induced gastrointestinal allergies highlights the variety of symptoms that may be experienced by patients. It also demonstrates the delay in getting this diagnosis recognised and highlights elimination diets that are being routinely used in a tertiary centre that has a specialist interest in food induced gastrointestinal allergies. However, further prospective studies in this area of food hypersensitivity are vital.

## Competing interests

This work has not been funded by any commercial funding or grant body, but has been performed through the National Health Service in the UK. None of the authors have declared any conflict of interest that may have impacted on the publication of this publication.

## Authors’ contributions

KA and LM carried out the data collection for the project. RM analysed the data and prepared the manuscript. AF, VC, NT, KL, KA, MA, GOD, LM assisted in interpreting the data and in the review of the manuscript. All authors read and approved the final manuscript.
